# Morpho-Anatomical Modulation of Seminal Roots in Response to Water Deficit in Durum Wheat (*Triticum turgidum* var. *durum*)

**DOI:** 10.3390/plants13040487

**Published:** 2024-02-08

**Authors:** Oum Cheikh Felouah, Faiza Ammad, Ahmed Adda, Assia Bouzid, Mohammed Lotfi Gharnaout, Philippe Evon, Othmane Merah

**Affiliations:** 1 Laboratory of Agro-Biotechnology and Nutrition in Semi-Arid Zones, Faculty of Nature and Life Sciences, University Ibn-Khaldoun, Tiaret 14000, Algeriaassia1bouzid@gmail.com (A.B.); 2 Laboratory of Plant Physiology Applied to Aboveground Crops, Faculty of Nature and Life Sciences, University-Ibn-Khaldoun, Tiaret 14000, Algeria; 3 Laboratoire de Recherché Protection et Valorisation des Produits Agrobiologiques, Departement de Biotechnologie, Faculté des Sciences de la Nature et de la vie, Université Blida-1, BP 270, Blida 09000, Algeria; ammad.faiza@yahoo.com; 4 Département Génie Biologique, IUT A, Université Paul Sabatier, 32000 Auch, France; mohammed.gharnaout@iut-tlse3.fr; 5 Laboratoire de Chimie Agro-industrielle (LCA), Université de Toulouse, INRAe, INPT, 31030 Toulouse, France; philippe.evon@etoulouse-inp.fr

**Keywords:** durum wheat, drought, seminal roots, morphology, anatomy, xylem vessel

## Abstract

The productivity of durum wheat in Mediterranean regions is greatly reduced by water deficits that vary in intensity and time of occurrence. The development of more tolerant cultivars is the main solution for fighting these stresses, but this requires prior study of their mechanisms. The involvement of the root system in drought avoidance is of major importance. It is in this context that the present work attempts to establish the impact of morpho-anatomical remodeling of seminal roots on dehydration avoidance at the javelina stage in five durum wheat genotypes grown under three water regimes, 100%, 60% and 30% of field capacity (FC). In the last two treatments, which were applied by stopping irrigation, moisture was concentrated mainly in the depths of the substrate cylinders and was accompanied by greater root elongation compared with the control. The elongation reached rates of 20 and 22% in the ACSAD 1231 genotype and 12 and 13% in the Waha genotype, in the 60% FC and 30% FC treatments respectively. The seminal roots anatomy was also modified by water deficit in all genotypes but to different degrees. The diameter of vessels in the late metaxylem vessels was reduced, reaching 17.3 and 48.2% in the Waha genotype in the 60% FC and 30% FC treatments, respectively. The water deficit also increased the number of vessels in the early metaxylem, while reducing the diameter of its conducting vessels. ACSAD 1361 and Langlois genotypes stood out with the highest rates of diameter reduction. The morpho-anatomical transformations of the roots contributed effectively to the plants’ absorption of water and, consequently, to the maintenance of a fairly high relative water content, approaching 80%.

## 1. Introduction

The environment generates different abiotic stresses that affect the development of plants and reduce their productivity. Thus, in several regions of the world, water deficits constitute one of the main stresses which strongly reduce the yield of rainfed crops. Among the latter, winter cereals, including durum wheat [1,2], are the most exposed to this environmental constraint. Such a situation prevails in Algeria, as in several Mediterranean regions, where cereal growing is mainly located in semi-arid areas [1,2]. In the case of Algeria, the area reserved for the cultivation of cereal species is mostly located in the high plateaus and inland plains of the country, which are characterized by a succession of drought periods of varying intensity. Thus, the water supply of plants is the main factor inducing quantitative and qualitative variations in these crops’ productivity. These conditions have been further exacerbated by the decrease in seasonal precipitation due to global warming that is becoming a reality [3]. Indeed, it has been observed recently that the frequency of drought declaration has increased significantly, particularly in autumn, which coincides with the early stages of the cereal cycle. The plant’s vulnerability during this period intensifies the effects of drought and compromises crop success [4]. This agronomic impact results from the constraints generated by water deficit at different levels of plant structure and function, and their environmental factors [5]. Indeed, a change in the soil’s physicochemical properties leads to a reduction and imbalance in the absorption of nutrients by the roots and their translocation to the plant’s various organs. Drought reduces growth, shortens cycles and disrupts the processes involved in developing yield components. Thus, drought causes cell dehydration, disorganizes membranes and various metabolic processes, and reduces energy transfer and photosynthetic yield, which has a negative impact on cell multiplication, elongation and differentiation, thus affecting plant growth and development [6].

In this situation, two strategies for combating drought are needed: the use of supplemental irrigation, which remains inaccessible for most farms, and or the use of cultivars that are more tolerant to the drought. Such tolerance can be achieved by drought avoidance or strain avoidance and their combination [7]. The processes involved in these strategies are complex and involve physiological, morphological and anatomical traits expressed by different plant organs [8,9].

Among these organs, roots play an essential role in water deficit tolerance [10,11,12]. Indeed, the morphological and structural remodeling of roots under drought, are greatly involved in plant dehydration avoidance [13,14,15].

The durum wheat plant, like all grasses, is characterized by the coexistence of two types of roots, seminal and adventitious. The early differentiation of the seminal roots confers on them the exclusive right to an assured water supply during the first stages of plant development [16,17].

The involvement of roots in the tolerance of plants to water deficit is largely based on their very sensitive perception of variations in substrate moisture and the fact that they show great structural and functional plasticity in response to these water availabilities [9,18]. Therefore, different types of remodeling can take place in the root system during the different phases of plant development to ensure a better efficiency of use of available water in the soil. Morpho-anatomical changes stand out as the main changes occurring under conditions of deficient water supply. Among these, increased root elongation is one of the key mechanisms allowing plants to access deeper and wetter soil layers under drought conditions [19,20]. This strategy is more necessary in Mediterranean climates characterized by alternating rainy and dry periods, resulting in the percolation of moisture into the deeper layers of cultivated soils. In fact, some work [21,22] suggests that under these conditions, rooting depth can be considered one of the key drought avoidance traits. This morphological change is inevitably be accompanied by anatomical transformations that need to be clarified. A prominent work in this field of research is that of Richards and Passioura [23], who reported that the reduction in the diameter of the late metaxylem vessels of the seminal roots of wheat increases their hydraulic resistance and improves the circulation of the sap within them. As a result, water deficit-tolerant wheat genotypes thus maintain higher water conductivity than susceptible genotypes [22,23,24,25,26]. More in-depth studies on the role of the morpho-anatomical remodeling of the seminal roots in the avoidance of stress related to water deficits are necessary. Thus, clarifying the role of these transformations in the tolerance to high water potential and the preservation of the water content of plants is the main objective of the present study. The main objective is to investigate the morphological and structural modifications of the seminal roots in five genotypes of durum wheat subjected to three water treatments, 100%, 60% and 30% of substrate field capacity (FC). Therefore, the growth in the length of the roots as a result of changes in water content along the profile of the culture substrate was evaluated. Also, the structural changes of the xylem conducting tissues were investigated. We have assessed the contribution of these transformations to the preservation of the water status of the tested plants.

## 2. Results

### 2.1. Soil Water Content

**Figure 1:**
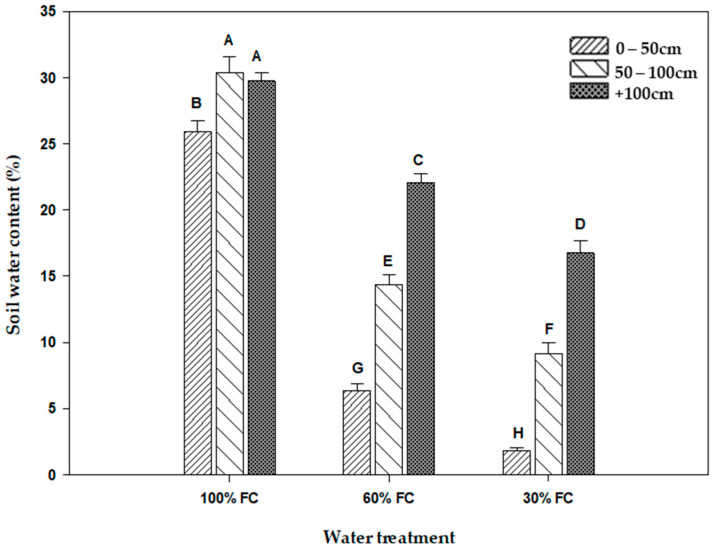
Soil water content (%) at three depths (0–50 cm, 50–100 cm, beyond 100 cm) in each water treatment (30% FC, 60% FC, 100% FC). Means indicated by different letters are significantly different (at 0.05 probability level) by Duncan’s test.

Significant differences in the moisture distribution at different depths of the cylinders was noted among the different water treatments. Thus, the soil moisture content increased from the surface to the depth (Figure 1). For the treatment of 30% FC, the moisture was concentrated mainly in the first 100 cm of the cylinder. Therefore, the water content of the soil, which is low at the surface, increases significantly with depth. The same moisture distribution in the cylinders was found in the 60% FC treatment. In fact, the water content increased with depth in the same way, except that the values were higher than in the 30% FC treatment. However, under the control treatment (100% FC), the water content of the soil, which was higher overall, was uniformly distributed among the three depths (Figure 1). Thus, its values are higher than 25%.

### 2.2. The Relative Leaf Water Content

The relative leaf water content of the tested plants was moderately affected by water supply variations (Table 1 and Table 2). Therefore, in the control treatment (100% FC), the average relative leaf water content was around 90.6%, while the maximum values were recorded for ACSAD 1361 and Oued Zenati with 91.5%, and the minimum value was observed in Waha with 89.5% (Table 2).

The reduction in the water content of the substrate at the level of 60% FC induced a weak decrease in the relative leaf water content. Therefore, Waha recorded the smallest reduction at 4.6%, while Langlois recorded the highest decrease at 6.4%. We note that under this water treatment, the values of the relative leaf water content varied between 84.6% and 87%, which were recorded in Mexicali 75 and Oued Zenati, respectively.

Lastly, in plants grown at 30% FC, the relative leaf water content was lower than that of the control, with the greatest difference estimated at −11.8%, which was noted in the Oued Zenati genotype. However, the relative leaf water content remained fairly high, with values ranging from 79.6% (Waha) to 81.7% (ACSAD 1361). The reductions in relative leaf water content that accompanied the lowering of the moisture content of the growing medium were small. In fact, the moisture values of this trait were maintained at levels above 75% in both treatments (60% FC, 30% FC) subjected to water deficit.

### 2.3. Seminal Root Length

Variations in water supply had a significant effect on seminal root length (Table 1). Indeed, the decrease in soil water content in the 60% FC and 30% FC treatments induced root elongation (Table 3). This modification affected all the studied genotypes, but to varying degrees. In the control treatment, average seminal root lengths varied between 123.5 and 141 cm, which were observed in ACSAD 1361 and Oued Zenati, respectively. In the 60% FC treatment, the maximum root length values ranged from 151.8 cm to 154.8 cm for the Langlois and ACSAD 1361 genotypes, respectively. In the same water treatment, genotype ACSAD 1361 recorded the highest root elongation rate at 20.2%. In contrast, the Langlois genotype recorded the lowest rate at 7.4%.

The moisture depression of the substrate in the treatment of 30% FC, resulted in genotypes responding with an even more pronounced rate of root elongation. The ACSAD 1361 genotype was distinguished by a high root elongation rate of 22.1% and a length of 158.5 cm.

It was found that genotypes that exhibited the shortest roots under the optimal water supply conditions (100% FC), had the highest root elongation rates under water deficit. This was the case for ACSAD 1361 and Waha, which recorded increases in root length of 20.2% and 22.1%, and 12.2% and 13% under treatments of 60% FC and 30% FC, respectively.

### 2.4. Anatomical Traits of Seminal Roots

The substrate water content and the water supply of plants promote changes in seminal roots anatomy. In fact, the results show that the anatomical parameters studied depend on water supply and genotype (Table 1). Indeed, decreased substrate water content resulted in significantly reduced diameters of late and early metaxylem vessels. However, the decrease in soil water content caused an increase in the number of early metaxylem vessels (Table 1).

#### 2.4.1. Late (Central) Metaxylem Vessel Diameter

The seminal roots of wheat are distinguished at the end of their differentiation by a central conductive element called the late metaxylem. It mainly ensures the sap transport. The diameter of these elements was greatly modified by the substrate water content (Table 4). In fact, the reduction in substrate water content was accompanied by a reduction in the diameter of the vessels of this xylem component. However, this effect was highly dependent on the nature of each genotype. In the control treatment (100% FC), vessel diameters ranged from 197 µm (Waha) to 223.6 µm (Oued Zenati), while in the treatment subjected to water deficit at 60% FC, the diameters decreased variably in all genotypes to reach extreme values of 184.6 µm and 214.7 µm, recorded for ACSAD 1361 and Oued Zenati, respectively. With the same treatment and concerning diameter reduction rates compared with the control, we note that the Waha genotype recorded the greatest reduction at 17.3%, while the smallest reduction of 1.2% was observed in Langlois. In the treatment carried out at 30% FC, the decreases in the diameter values were even more important where they reached rates of 48.2% and 23.4% for the two genotypes Waha and Oued Zenati, respectively. In this same water treatment (30% FC), the metaxylem diameter of Langlois seemed to be the least sensitive to water supply variations, recording the smallest reduction of 10.2%. In this treatment, the vessel diameters were delimited by mean values of 102 and 186.9 µm, recorded in Waha and Langlois, respectively.

#### 2.4.2. Number and Diameter of Early Metaxylem Vessels

**Figure 2:**
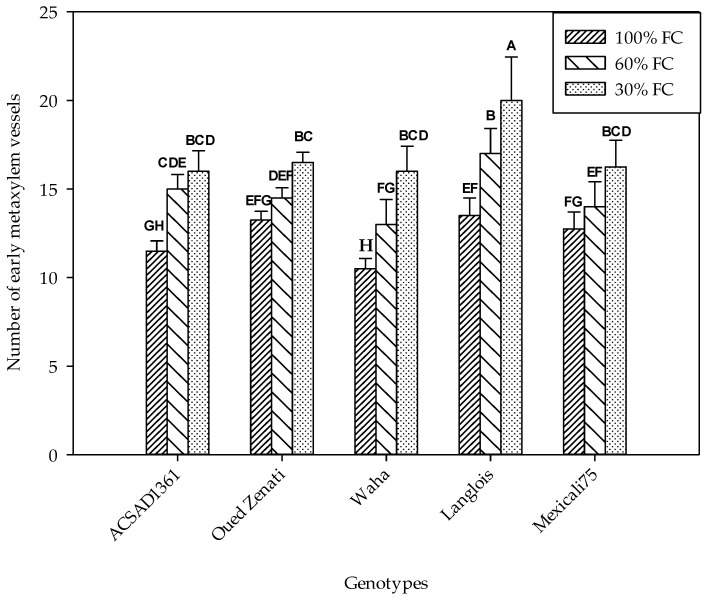
Number of early metaxylem vessels measured on five durum wheat genotypes grown under three water regimes (100% FC, 60% FC, 30% FC). Values of genotypes within the same water treatment and assigned different letters are significantly different (at 0.05 probability level) by Duncan’s test.

The number of vessels of the early metaxylem was significantly dependent on the water regime as well as on the nature of the genotypes (Table 1). In fact, it increased progressively with decreasing soil water content (Figure 2). In the control treatment, the average number ranged from 10.5 to 13.5, which were found in the Waha and Langlois genotypes, respectively. Under water deficit conditions at a level of 60% FC, these values were of the order of 13 and 17 for the same genotypes. Finally, in the treatment carried out at 30% FC, only Langlois stood out among all the genotypes with 20 metaxylem vessels, whereas Waha recorded an average value of 16.

**Figure 3:**
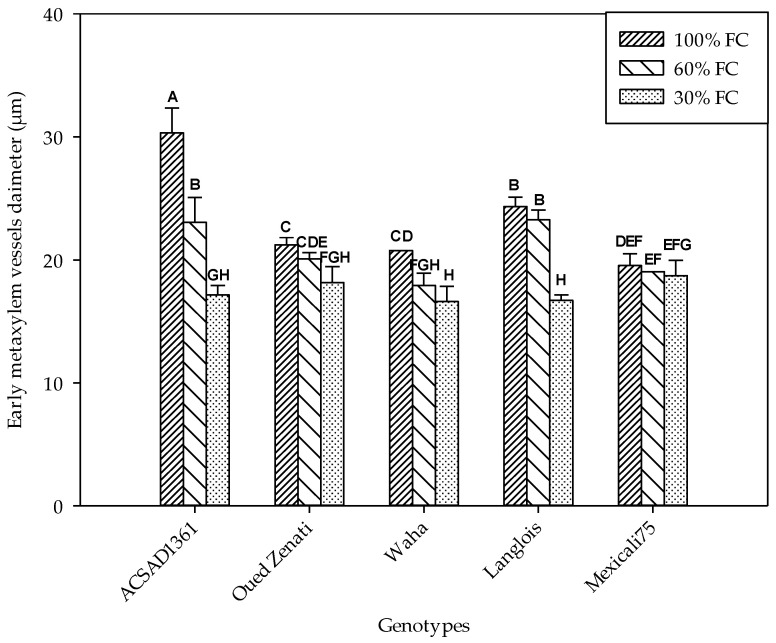
Early metaxylem vessel diameter (µm) measured on five durum wheat genotypes grown under three water regimes (100% FC, 60% FC, 30% FC). Values of genotypes within the same water treatment and assigned different letters are significantly different (at 0.05 probability level) by Duncan’s test.

The variation in the diameters of the early metaxylem conducting vessels was significantly influenced by genotype and water treatment (Table 1). A significant interaction effect of these two factors was also found, indicating that its development is a genotypic distinction. Therefore, the decrease in soil water content was accompanied by a decrease in the diameter of these vessels (Figure 3). In the control, ACSAD 1361 distinguished itself by having the widest vessels with an average value of 30.3 µm, while Mexicali 75 showed the narrowest at 19.5 µm. The reduction in substrate moisture in the 60% FC treatment was accompanied by a decrease in vessel diameter, with mean values ranging from 23.3 µm (Langlois) to 17.9 µm (Waha). The reduction was even greater in the 30% FC treatment, where diameter values ranged from a minimum of 16.6 µm to a maximum of 18.7 µm in the Waha and Mexicali 75 genotypes, respectively.

## 3. Discussion

The relative leaf water content (RWC) is an effective indicator for assessing plant water status. It, therefore, reflects the water supply status of plants [27,28,29,30]. Several studies [31,32] have shown that during drought, the perception of stress initially results in a decrease in relative leaf water content. However, other studies [31,32,33,34] have reported that a plant’s tolerance to water deficit is reflected in its ability to maintain an optimal hydration state and maintaining relative leaf water content at a high level. The results obtained in the present study show that the application of water deficit in the 60% FC and 30% FC treatments was accompanied by a small proportional reduction in relative leaf water content. Indeed, the average relative reduction in water content reached 5.4% and 11.2% in the 60% FC and 30% FC treatments, respectively. It was maintained at levels above 84% and 79% in the two treatments subjected to water deficit. Maintaining optimal hydration of plants under water deficit is ensured mainly by water absorption efficiency [35,36]. Thus, the morpho-anatomical remodeling of roots occurring under water deficit plays a major role in this strategy [37,38].

The results obtained in the present study show that the lowering of the water content of the culture substrate caused profound morpho-anatomical changes in the seminal roots, which could contribute to the avoidance of water stress. Thus, the decrease in water content in the culture cylinders was associated with an increase in seminal root length, which reached rates of 11.2% and 12.8% in the 60% FC and 30% FC treatments, respectively. This root elongation occurred in order to expose the absorbent zone of the roots to the wetter substrate layers that were presumably concentrated at the bottom of the cylinders (Table 1). The results of studies on drought effects on seminal root morphology in durum wheat are sometimes contradictory and depend on the conditions under which the experiments were conducted. Studies on durum wheat [39] and maize reported that soil drying favored root elongation. In contrast, root shortening was observed by Bchini et al. [40] in wheat and by Thomas et al. [41] in barley.

The root elongation caused by the water deficit (60% FC, 30% FC) allowed an increase in the water absorption capacity as roots reached the deeper horizons of the soil, which remain more moist [17,42]. This effect was demonstrated when the water deficit was caused by stopping irrigation and the drying of the soil extended from the surface to depth. The increase in root length under water deficit observed in this study allowed the plants to reach the bottoms of the cultivation cylinders, which were wetter, and access an optimal water supply, allowing them to maintain high relative leaf water content. The progressive drying of the substrate from the surface results in greater moisture at depth, which modulates root growth length and has been described as hydrotropism in some works [12,43].

Variations in soil water content also cause significant changes in the structure of the absorbing piliferous zone of seminal roots. These changes are more pronounced in a water deficit situation. They were mainly expressed in the treatment conducted at 30% FC. They mainly concern the structure of the xylem conducting vessels. The xylem of the seminal root of wheat consists of several early conducting vessels and a single late (central) vessel. They differ in their diameter, with the central vessel being much larger. The results show that the decrease in soil water content caused a significant decrease in the diameter of vessels in all the genotypes tested, to varying degrees. This reduction was greater in the 30% FC treatment where the average reduction observed was 22.4%. These results are confirmed by other studies which have shown that drought induces a drop-in leaf water potential due to a reduction in the number and diameter of xylem conducting vessels [44,45]. These transformations enable the continued flow and ascent of sap under water deficit conditions. Thus, some studies, including on rice [28], seed legumes [26] and wheat [9], have shown that anatomical transformations of the roots related to cellular compaction significantly improve the tolerance to water deficit of these species. The reduction in the diameter of the conducting vessels in the central cylinder, which results in a reduction in the root diameter, does not affect the water absorption efficiency [46].

Our results indicate that the water deficit in the treatment performed at 30% FC caused an increase in the number of the early metaxylem by an average rate of 27.4%. This effect was greatest for the local Langlois genotype, which is known for its great adaptation to water deficits. On the other hand, the decrease in substrate moisture caused a decrease in the diameter of the conductive elements of this tissue, with an average decrease of 24.8% in the treatment conducted at 30% FC. The transformations that affect the conducting vessels of the metaxylem are part of a compensation strategy in the efficiency of xylem sap transport, the flow of which is closely related to the availability of water and the plant’s capacity for absorbing it. In fact, under waterlogging conditions, the opposite situation is observed, with low root length growth which generates an essentially superficial root density. Also, the structure of these roots is characterized by fairly high cell growth, resulting in the formation of roots with a large diameter and larger xylem conducting vessels [47].

Reducing the diameter of late metaxylem vessels increases the capacity for the upward transport of the sap, the quantity of which is compensated by an increase in the number of peripheral conducting elements. The strategy thus developed by the plants to avoid water deficit involves the exploration of moisture in the depth of the cylinders through root elongation, and a better ascent of the sap is ensured by the structural transformations of these organs. These traits will be retained as selection criteria as long as the variability expressing them is present in this species.

## 4. Materials and Methods

### 4.1. Plant Material and Experimental Design

Five durum wheat genotypes (*Triticum turgidum* var. *durum*) were used in our study. These included two local landraces (Oued Zenati, Langlois) and three other advanced lines, Waha, ACSAD 1361 and Mexicali 75, introduced from ICARDA (International Center for Agricultural Research in the Dry Areas, Aleppo, Syria), ACSAD (Arab Center for the Study Arid Zones and Dry Lands) and CIMMYT (International Maize and Wheat Improvement Center), respectively. The main agronomic traits of the genotypes tested are presented in Table 5.

The experiment was carried out in a greenhouse with controlled conditions at the Faculty of Natural and Life Sciences of the University -Ibn-Khaldoun of Tiaret (34°04′ North and 1°33′ East), Algeria. Diurnal and nocturnal temperatures were maintained at 25 °C and 15 °C, respectively. The seeds were disinfected with sodium hypochlorite, rinsed with distilled water and sown in PVC cylinders (150 cm height and 5 cm diameter) filled with a mixture of soil, sand and organic matter (8:3:1), with a water retention capacity of 27%. All the cylinders were irrigated to the field capacity and after emergence of the first leaf, the three water treatments were applied. Control plants were grown in soil constantly held at 100% field capacity (FC). For the other treatments, we proceeded gradually in withholding water so that at the end of the experiment the treatment moisture levels were obtained (60% and 30% FC). Irrigation management to obtain 60% and 30% FC was established by calculating the weights that the cylinders of these two treatments should have, while knowing the water retention capacity of the substrate. Initially, the three treatments were irrigated at field capacity (100% FC), and then the amount of water lost through evapotranspiration per unit time was determined by weighing the cylinders. Based on the amount of water lost, we then determined the number of days required to reach substrate water contents in accordance with the set quantities (30% FC, 60% FC). The water contents of three control cylinders of each treatment were also determined at the end of the experiment. Each genotype was replicated four times in each water treatment, and the cylinders were randomly arranged within the treatments.

### 4.2. Traits Measurements

#### 4.2.1. Relative Leaf Water Content

The relative leaf water content was determined according to the method developed by Slatyer [48]. The fourth completely differentiated leaf was excised and immediately weighed to obtain the fresh weight (*FW*). The cut base was then immersed in distilled water and placed in the dark at 4 °C for 12 h and reweighed to determine the weight in full turgor (*FTW*). The dry weight (*DW*) was then determined at 80 °C after 48 h, and the relative leaf water content was calculated according to the following formula:

$$RWC\;\left(\%\right)=\frac{\left(FW-DW\right)}{FTW-DW}\times 100$$

The rate of change of the trait values, decreasing or increasing, was calculated according to the following formula:

$$Evolution\;\left(\%\right)=\frac{\left(VTC-VTT\right)}{VTC}\times 100$$

where *VTC* is the trait value in the control treatment, and *VTT* is the trait value in the 30% FC or 60% FC treatment.

#### 4.2.2. Morphological and Anatomical Traits of Seminal Roots

At the end of the experiment, at the tillering stage, the cylinders were emptied of their contents by abundant washing with a water jet. The roots of 60 plants from the four replicates of each genotype within each water treatment were recovered. The length of the axis of the seminal roots was determined using a 2 m long graduated tape. To obtain histological sections, a 1 cm section of the piliferous zone of the seminal roots was removed and fixed in a mixture of ethanol, formaldehyde and acetic acid (17:3:1) for 24 h. The samples were then washed and dehydrated in ethanol at increasing concentrations (50°, 70°, 90° and twice at 100°) for 15 min for each bath. The samples were embedded in warm paraffin after passing through baths of pure toluene and toluene/paraffin (50%:50%). After cooling of the paraffin blocks, cross sections of 7 µm thickness were obtained by a microtome (Leica RM 2125 RT). The sections were glued with gelatin on slides, dewaxed in three baths of toluene, rehydrated with ethanol at a concentration of 50%, and colored with alum carmine and methyl green. The sections were then dehydrated in two 100° ethanol baths, passed through a toluene bath, and protected with Canada balsam on top of coverslips. Observations and measurements were made by a microscope (Leica B-350) equipped with micro image analysis software OPTIKA (OPMIAS Ver. 1.0). Measurements were obtained for the diameter (µm) of the central or late metaxylem vessels, and the number and diameter (µm) of early metaxylem vessels. The morphological and anatomical traits were assessed on four replicates for each genotype in each water treatment.

#### 4.2.3. Soil Water Content

At plant harvest, soil samples from the three water treatments were collected in order to evaluate the soil water content at three depths (0–50 cm, 50–100 cm, beyond 100 cm). Soil samples were weighed and then reweighed after drying at 105 °C for 24 h. The soil water content (*SWC*) was determined according to the following formula:

$$SWC\;\left(\%\right)=\frac{SW-DRW}{SW}\times 100$$

where *SW* is the soil weight, and *DRW* is the dry soil weight.

The measurements were performed on four samples for each depth in each water treatment.

### 4.3. Statistical Analyses

All data were subjected to analysis of variance using the factorial ANOVA of Statistica software (V.8.0, USA). The effects of genotype, water treatments and their interaction on the studied traits were determined. Means comparison was performed using Duncan’s test at 0.05 probability level.

## 5. Conclusions

With frequent drought declarations occurring in several Mediterranean cereal-growing zones and with these conditions being further accentuated by climate change, it is essential to initiate studies to define plant tolerance strategies to drought stress. The results of our study showed that avoidance mechanisms represent a highly effective strategy for durum wheat against the effects of drought. Indeed, the maintenance of optimal hydration of wheat plants in water-deficit situations was supported by morpho-anatomical remodeling of roots during an early phase of their development. Responses through elongation of seminal roots enabled the most water deficit-tolerant genotypes to explore deeper, wetter layers of the soil. Structural changes in the roots revealed a narrowing of the central and peripheral xylem vessels, which is thought to improve the efficiency of sap ascent under water-deficient conditions. The results of the present study highlight the distinct behavior of the genotypes tested in relation to water deficit. The two local genotypes, Oued Zenati and Langlois, showed adaptation due to small variations in the parameters studied among different water treatments. In contrast, the two introduced genotypes, Waha and ACSAD 1361, showed tolerance by avoidance, due to strong variations in the expression of the mechanisms studied among the three water treatments. Root elongation and reduced xylem vessel diameter contribute to preserving plant hydration and are criteria for creating variability in durum wheat. These results need to be confirmed on a larger collection of genotypes before they can be used in breeding programs.

## Figures and Tables

**Table 1:** F-Test values and the significance of genotype, water treatment and their interaction effects on relative leaf water content, seminal root length and seminal root anatomical traits measured on five durum wheat genotypes cultivated under three water treatments.

Trait	Effect		
	Genotype	Water Treatment	Genotype × Water Treatment
	(d.f. = 4)	(d.f. = 2)	(d.f. = 8)
Relative leaf water content	4.3 **	247.7 ***	0.4 ^ns^
Seminal roots length	6.7 ***	392.8 ***	16.1 ***
Late metaxylem vessels diameter	4370 ***	10406 ***	961 ***
Early metaxylem vessels number	15.126 ***	74.586 ***	1.756 ^ns^
Early metaxylem vessels diameter	42.78 ***	143.48 ***	22.28 ***

*** Significant at *p* ≤ 0.001; ** significant at *p* ≤ 0.01; ^ns^ not significant.

**Table 2:** Mean values (±SE) of relative leaf water content (%) of five durum wheat genotypes and their decrease rate for the three water treatments (100% FC, 60% FC, 30% FC). Means indicated by different letters are significantly different (at 0.05 probability level) by Duncan’s test.

Genotypes	Water Supply				
	100% FC	60% FC	Decrease Rate (%)	30% FC	Decrease Rate (%)
ACSAD 1361	91.5 ^A^ ± 0.4	86.3 ^BC^ ± 0.41	5.6	81.7 ^D^ ± 0.82	10.7
Oued Zenati	91.5 ^A^ ± 0.31	87.0 ^B^ ± 0.26	4.9	80.7 ^D^ ± 0.73	11.8
Waha	89.5 ^A^ ± 0.61	85.3 ^BC^ ± 0.63	4.6	79.6 ^D^ ± 1.25	11.1
Langlois	90.8 ^A^ ± 0.98	84.9 ^BC^ ± 0.56	6.4	80.4 ^D^ ± 1.26	11.4
Mexicali 75	89.6 ^A^ ± 0.73	84.6 ^C^ ± 0.29	5.6	79.9 ^D^ ± 0.47	10.9
Means	90.56	85.63	5.43	80.44	11.17

**Table 3:** Mean values of seminal roots length (cm) of five durum wheat genotypes and their rate of increase among the three water treatments (100% FC, 60% FC, 30% FC). Means indicated by different letters are significantly different (at 0.05 probability level) by Duncan’s test.

Genotypes	Water Supply				
	100% FC	60% FC	Increase Rate (%)	30% FC	Increase Rate (%)
ACSAD 1361	123.5 ^F^ ± 2.9	154.8 ^A^ ± 1.6	20.2	158.5 ^ABC^ ± 0.9	22.1
Oued Zenati	141.0 ^D^ ± 0.7	153.3 ^AB^ ± 0.6	8.0	156.3 ^BC^ ± 1.3	9.7
Waha	135.3 ^E^ ± 0.5	154.0 ^ABC^ ± 0.9	12.2	155.5 ^BC^ ± 1.2	13.0
Langlois	140.5 ^D^ ± 0.8	151.8 ^ABC^ ± 1.0	7.4	155.8 ^C^ ± 1.8	9.8
Mexicali 75	140.5 ^D^ ± 0.5	153.3 ^ABC^ ± 1.0	8.3	155.3 ^BC^ ± 1.1	9.5
Means	136.15	153.40	11.2	156.25	12.8

**Table 4:** Late metaxylem vessels diameter (µm) measured on five durum wheat genotypes grown under three water regimes (100% FC, 60% FC, 30% FC). Values of genotypes within the same water treatment assigned different letters are significantly different (at 0.05 probability level) by Duncan’s test.

Genotypes	Water Supply				
	100% FC	60% FC	Decrease Rate (%)	30% FC	Decrease Rate (%)
ACSAD 1361	206.6 ^D^ ± 0.5	184.6 ^H^ ± 1.3	10.6	169.2 ^K^ ± 0.7	18.1
Oued Zenati	223.6 ^A^ ± 0.3	214.7 ^B^ ± 0.2	4.0	171.2 ^J^ ± 0.1	23.4
Waha	197.0 ^F^ ± 0.7	162.9 ^L^ ± 0.6	17.3	102.0 ^M^ ± 0.8	48.2
Langlois	208.2 ^C^ ± 0.4	205.6 ^D^ ± 0.3	1.2	186.9 ^G^ ± 1.1	10.2
Mexicali 75	200.0 ^E^ ± 0.1	187.0 ^G^ ± 0.4	6.5	175.9 ^I^ ± 0.4	12.0
Means	207.07	190.97	7.93	161.03	22.41

**Table 5:** The origin and main agronomic traits of tested genotypes.

Genotype	Origin	Drought Tolerance	Cycle Length	Stem Height
Oued Zenati	Algerian Landrace	Medium	Late	Tall
Langlois	Algerian Landrace	High	Late	Tall
Waha	ICARDA	High	Early	Dwarf
ACSAD 1361	ACSAD	Low	Early	Dwarf
Mexicali 75	CIMMYT	Medium	Early	Dwarf

## Data Availability

The data presented in this study are available upon request.
